# Random Neural Network Based Epileptic Seizure Episode Detection Exploiting Electroencephalogram Signals

**DOI:** 10.3390/s22072466

**Published:** 2022-03-23

**Authors:** Syed Yaseen Shah, Hadi Larijani, Ryan M. Gibson, Dimitrios Liarokapis

**Affiliations:** 1 School of Computing, Engineering and Built Environment, Glasgow Caledonian University, Glasgow G4 0BA, UK; ryan.gibson@gcu.ac.uk (R.M.G.); dimitrios.liarokapis@gcu.ac.uk (D.L.); 2 SMART Technology Research Centre, Glasgow Caledonian University, Cowcaddens Road, Glasgow G4 0BA, UK

**Keywords:** machine learning, epilepsy, random neural network

## Abstract

Epileptic seizures are caused by abnormal electrical activity in the brain that manifests itself in a variety of ways, including confusion and loss of awareness. Correct identification of epileptic seizures is critical in the treatment and management of patients with epileptic disorders. One in four patients present resistance against seizures episodes and are in dire need of detecting these critical events through continuous treatment in order to manage the specific disease. Epileptic seizures can be identified by reliably and accurately monitoring the patients’ neuro and muscle activities, cardiac activity, and oxygen saturation level using state-of-the-art sensing techniques including electroencephalograms (EEGs), electromyography (EMG), electrocardiograms (ECGs), and motion or audio/video recording that focuses on the human head and body. EEG analysis provides a prominent solution to distinguish between the signals associated with epileptic episodes and normal signals; therefore, this work aims to leverage on the latest EEG dataset using cutting-edge deep learning algorithms such as random neural network (RNN), convolutional neural network (CNN), extremely random tree (ERT), and residual neural network (ResNet) to classify multiple variants of epileptic seizures from non-seizures. The results obtained highlighted that RNN outperformed all other algorithms used and provided an overall accuracy of 97%, which was slightly improved after cross validation.

## 1. Introduction

Epileptic disorders result in seizures caused by abnormal brain activity. Epilepsy is a brain-centered central nervous system disorder that manifests itself in a variety of ways including loss of awareness and confusion. These symptoms result in fall injuries and patients often bite their tongues. Seizures are difficult to detect for researchers since they occur without warning. Epileptic seizures can be detected by observing electroencephalogram (EEG) signals of a patient. An EEG is a graphical record of the brain’s electric activity that indicates the voltage fluctuations in electrical activity recorded using multiple electrodes placed on various brain locations [1]. Clinical EEGs are used by doctors to monitor different types of epileptic activity since it leaves discrete imprints in the form of interictal epileptic discharges, high-frequency oscillations, and pre-ictal activities [2]. EEG signals have non-stationary and non-Gaussian characteristics that are used to measure the electrical activity of the brain to diagnose the type of brain disorder. An EEG is especially effective when it comes to studying the function of the brain and diagnosing epilepsy. EEG signal analysis can help distinguish between signals associated with epileptic episodes and normal signals.

The rapid development in the fields of the Internet of Things (IoT) and artificial intelligence (AI) has paved the way for improvements in various fields of life [3,4]. Over the past few decades, these technologies have also transformed the healthcare sector by lowering costs and increasing efficiency. Patients suffering from epilepsy sometimes have to depend on caregivers, assisted living, or nursing homes [5]. A system that could detect epileptic seizures automatically in a closed-loop environment could help healthcare centres monitor patients with epilepsy and detect epileptic seizures/events by employing technologies such as EEGs and electrocardiograms (ECGs). It could also potentially increase the efficiency of seizure detection and reduce the labor and effort needed for monitoring and analyzing the seizure patterns by a neurophysiologist with their naked eye.

Classification algorithms, such as those employed in this research, could help predict whether or not a person will have a seizure. Researchers have carried out extensive studies to understand electrical activities and dynamic properties of the brain to identify nonlinear deterministic dynamics found in seizure activity [6]. Furthermore, EEG-based brain activity has been analyzed using nonlinear time series analysis (NTSA) in the literature [7,8,9]. EEGs have been used in many studies that involved patients with various diseases, such as Alzheimer’s [10], depression [11], and Parkinson’s disease [12]. In addition, studies have been carried out on EEGs of patients with epilepsy [12,13,14,15] as well as those of healthy individuals [16,17,18]. The findings from epilepsy-related publications are classified into two categories: epileptic seizures [13,15] and brain activities of healthy individuals [19]. Researchers have investigated and modeled the nonlinearity of human brain behavior using these two categories. Moreover, the studies suggested that even minor changes in the dynamic system parameters of the brain can result in a range of physiological brain states [20,21] and may cause brain abnormalities [11,22,23] or other issues [6,24]. Furthermore, detection of epileptic seizures remains a source of consternation for many researchers [6,8,13,15].

This study aimed to develop a random neural network (RNN)-based deep learning model for the detection of epileptic seizures that utilized statistical features extracted from a publicly available dataset containing labeled data of three classes of different kinds of epileptic seizures, along with one class of normal data recorded from six patients. The main contributions of this study are summarized as follows:

- A novel AI-based classification model for automatic detection of epileptic seizures using an RNN algorithm was developed. The total number of classes include a normal and three different types of epileptic seizures, including complex partial, electrographic, and video-detected seizures with no visual change.
- The novel model was compared with that of other classification algorithms such as the convolutional neural network (CNN) and residual neural network (ResNet) considering all four classes, including normal and the three other types of seizures.
- A critical analysis of the results obtained demonstrated that the model was the most suitable classification method for the identification of various types of epileptic seizures.

The rest of the paper is organized as follows: Section 2 discusses recent state-of-the-art studies on epileptic disorders, including different machine learning algorithms and datasets used in the literature; Section 3 provides details of the adapted EEG-based dataset and a description of RNN, ResNet, ERT, and CNN; the experimental results are discussed in Section 4; and finally, Section 5 concludes the paper.

## 2. Related Work

Many studies have been carried out over the past few decades in order to better understand epilepsy and the brain activity related to epileptic episodes and to predict seizures. In [25] wavelet filters were used to restrict signals under 60 Hz. Various techniques, such as independent component analysis (ICA), were used to remove artifacts from EEG data and the data were segregated using blind source separation. In [26] the authors proposed a technique for the characterization of EEG time series data to improve the signal classification. Fotiadis et al. [27] performed classification of EEG segments for epilepsy using time-frequency analysis and compared various other methods for EEG analysis. The power spectrum density of each segment was calculated using short-time Fourier transform. In [28], the authors proposed a method for removing artifacts from EEG data to detect seizures in epileptic patients. After artifacts were removed from EEG data, the patterns that represented seizures in EEGs were easily distinguishable from non-seizure epochs. Epileptic seizures were detected by applying various entropy estimators to EEG signals recorded from healthy individuals and epileptic patients in [29,30,31]. The authors in [32] used the eigenvector method for feature extraction, and trained the classifier using the extracted features from the EEG data.

Several methods for the prediction of epileptic seizures have been proposed in the past few years; however, none of them have relied on a single metric as a measure for the performance of the proposed method. Moreover, researchers have used a variety of publicly available datasets in their studies and the different datasets might also have affected the performance of a specific classification model. Therefore, while evaluating the performance of a classification model the dataset used should also be taken into account.

## 3. Methods and Materials

### 3.1. EEG Dataset for Epilepsy

The dataset, which is publicly available, was recorded at the American University of Beirut. The dataset contains EEG recordings of 6 patients suffering from focal epilepsy. The patients were being evaluated for possible epileptic surgery with long-term EEG-video monitoring. The dataset consists of multi-channel time-series data recorded at a sampling rate of 500 Hz stored in European data format (EDF), which stores header information, as well as raw data that contain 19 channels where each channel represents an electrode placed on the patient’s scalp. There are a total of 35 seizures recorded, and they are divided into 3 categories: 1. complex partial seizures; 2. electrographic seizures; and 3. video-detected seizures with no visual change over EEG.

The data were pre-processed and labelled as normal data and data that contain epileptic seizures. Two of the channels (Cz and Pz) were dropped in order to have uniform data across all records. The classified data were stored in matrices and each matrix had a size of 19 × 500 that stored 1 s of data containing 19 channels and 500 samples. The data were labelled as 0, 1, 2, and 3 where label 0 represented normal data containing 3895 s of information. The labels 1, 2, and 3 represented records with epileptic seizures representing complex partial seizures, electrographic seizures, and video-detected seizures containing 3034, 705, and 111 seconds of data, respectively. The number of matrices labelled 0 (normal data) was 3895, while the number of those matrices with labels 1, 2, or 3 was also 3895 combined. The number of samples for seizure and non-seizure data were kept the same intentionally so that the data were balanced for machine learning. The complete description of the dataset can be found on the Mendeley data platform [33].

### 3.2. Random Neural Network-Based Epilepsy Prediction Scheme

Erol Gelenbe first introduced RNN in his groundbreaking work in 1989 [34]. RNNs have been widely used as a classification algorithm in a variety of fields such as energy conservation [35], intrusion detection in IoT [36], and fall detection [37,38]. However, the effectiveness of RNN has not been reported in the literature for the prediction of epilepsy.

In RNN, a neuron triggers an excitatory and inhibitory state whenever it receives a signal with positive or negative potential. The accumulated signal received is represented by a non-negative integer that describes the potential state of the neuron. Neurons contained in RNN layers exchange excitatory and inhibitory signals in a probabilistic manner. When a neuron receives a +1 signal, it enters an excitation state, and a −1 signal represents an inhibition signal. The spiking signals are sent between neurons as impulses. A vector of non-negative integers $k_{l}\left(t\right)$ represents the potential state of a neuron *i* at time *t*. When $k_{l}\left(t\right)\leq 0$, a neuron is considered idle. Similarly, a neuron is considered to be excited if $k_{l}\left(t\right)>0$. A neuron can send a spike signal in the excited state at a rate of $r\left(l\right)\geq 0$, which reduces its potential by 1. The activation function $f_{l}$ can be expressed mathematically as follows.

(1) $$f_{l}=\frac{\lambda_{l}^{+}}{r_{l}+\lambda_{l}^{-}}$$

where excitatory and inhibitory inputs are $\lambda_{l}^{+}$ and $\lambda_{l}^{-}$, respectively:

(2) $$\lambda_{l}^{+}=\sum_{j=1}^{N}f_{j}r_{j}p_{j,l}^{+}$$

and

(3) $$\lambda_{l}^{-}=\sum_{j=1}^{N}f_{j}r_{j}p_{j,l}^{-}$$

The firing rate of neuron *j* is $r_{j}$, and $r_{l}$ is the firing rate of neuron *l*. The probability of whether a signal transmitted from neuron *j* to neuron *l* is excitatory or inhibitory is $p_{j,l}^{+}$ or $p_{j,l}^{-}$, respectively.

The activation function $f_{l}$ can also be expressed as follows:

(4) $$f_{l}=\frac{\sum_{j=1}^{N}f_{j}r_{j}p_{j,l}^{+}}{r_{l}+\sum_{j=1}^{N}f_{j}r_{j}p_{j,l}^{-}}$$

Also,

(5) $$\sum_{j=1}^{N}\left[p_{l,j}^{+}+p_{l,j}^{-}\right]+d\left(l\right)=1,\forall_{l}$$

Here, the probability of a signal leaving the network is represented by d(l). In (5), *N* represents the total number of neurons. The sum of all probabilities in the network must be equal to 1. When a neuron is in the excited state, the rate of positive and negative spikes it sends is expressed as follows:

(6) $$w^{+}\left(l,j\right)=r_{l}p_{l,j}^{+}\geq 0,$$

(7) $$w^{-}\left(l,j\right)=r_{l}p_{l,j}^{-}\geq 0,$$

where $r_{l}$ represents the firing rate of neuron l, which is derived by Gelenbe in [34] from Equations (5)–(7),

(8) $$r_{l}={\left(l=d\left(l\right)\right)}^{-1}\sum_{j=1}^{N}\left[w^{+}\left(l,j\right)+w^{-}\left(l,j\right)\right]$$

The weights $\;w^{+}\left(l,j\right)$ and $w^{-}\left(l,j\right)$ are similar to the weights in a classical neural network, according to Gelenbe [34], and traditional optimization algorithms such as gradient descent may be used to train the neural network.

**Figure 1:**
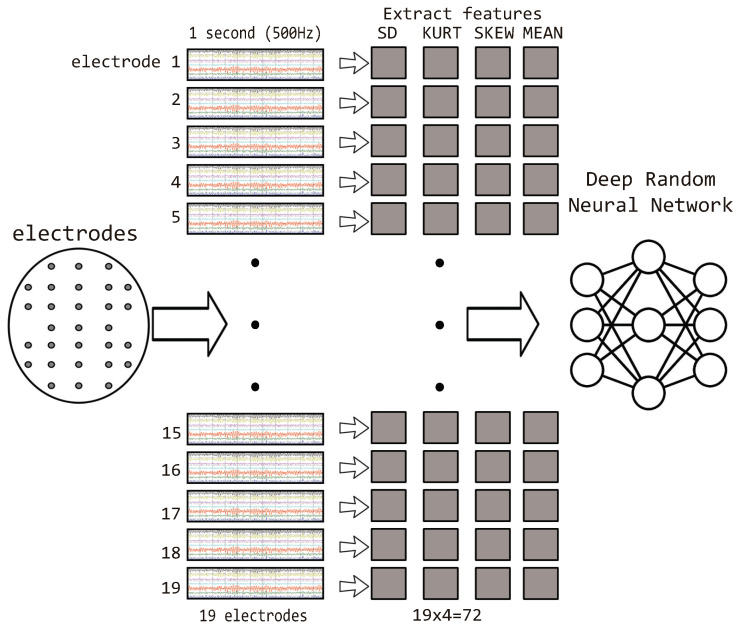
EEG-focused data pre-processing and feature extraction using the RNN algorithm.

In this study, a novel RNN-based deep learning model was proposed for the multi-class classification of epilepsy data. The data were obtained from a publicly available dataset [33]. Each sample in the dataset contained 19 channels, and each channel represented a data stream coming from a single electrode with a sampling rate of 500 Hz. The total number of samples in the training and testing data was 7011 (90%) and 779 (10%), respectively. In the pre-processing phase, features (standard deviation, kurtosis, skewness, and mean) were extracted from every 1 s segment of each electrode, as shown in Figure 1. The extracted features, i.e., standard deviation, kurtosis, skewness, and mean, have been used in literature for the classification of EEG data of patients with Alzheimer’s disease and mild cognitive impairment [39] achieving 95% accuracy and epilepsy [40] with an accuracy of 99.7%.

The features were normalized by scaling the numbers between 0 and 1 using the equation below:

(9) $$X_{normalized}\;=\;\frac{X\;-\;X_{min}}{X_{max}\;-\;X_{min}}$$

**Figure 2:**
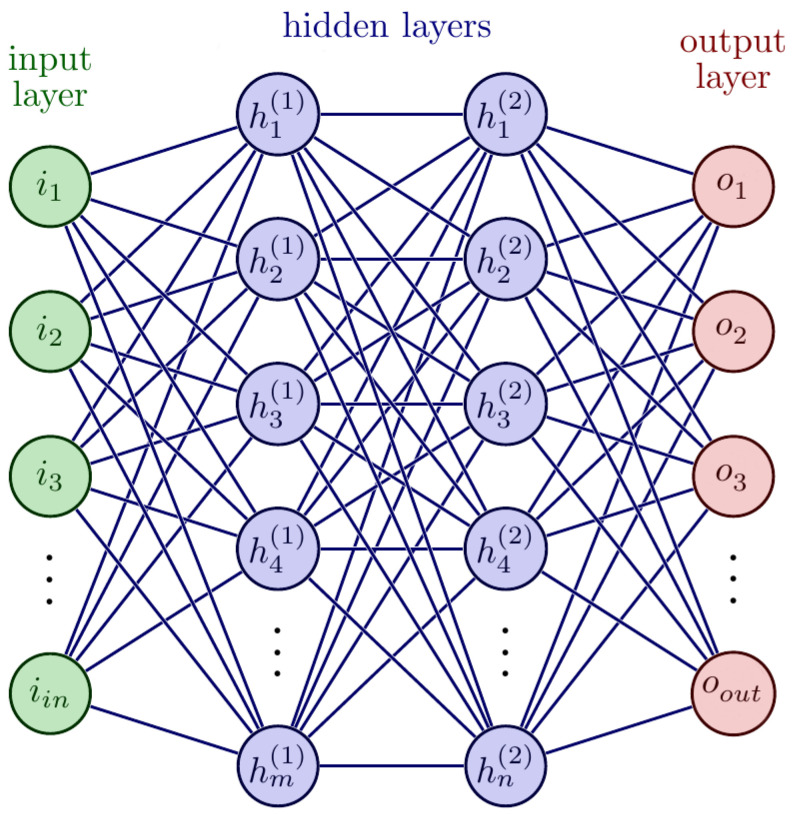
Deep RNN model for different types of epilepsy detection and classification.

Classification of the data was performed using the RNN model, as shown in Figure 2. The input layer consisted of 76 nodes, which was the number of values acquired in each sample after pre-processing. After optimizing the network values for the hidden layer, as depicted in Figure 2, the total number of neurons in hidden layers was 100 and 80, respectively. Since there were a total of four data classes, the number of nodes at the output layer was kept at 4.

### 3.3. Convolutional Neural Network-Based Epilepsy Detection

Due to its ability to learn features in images and excellent classification results on data with multiple classes, CNN has been proven to be ideal for image-based data. Researchers have shown a lot of interest in recent years in CNN for image recognition and it has been used in the analysis of medical X-ray images [41], magnetic resonance imaging (MRI) for brain tumor segmentation [42], and histopathological images for cell segmentation [43]. However, there has been limited research on the use of CNN with physiological signals. A CNN is made up of a convolution layer (Conv) and a pooling layer (Pool) with a rectified linear unit (ReLU) activation function, as well as batch normalization. It also has fully connected, drop-out, softmax, and classification output layers in the final layers. Filters in the Conv layer recognize various features in an image such as edges, objects, and shapes.

Table 1 shows the architecture of a CNN used for the classification of epilepsy data. The first layer of the proposed model was a two-dimensional convolutional layer (Conv2D), which takes a three-dimensional array (19 × 500 × 1) as input. Instead of an image, the input data contained 1 second of raw data with a sample rate of 500 from 19 channels of electrodes. Moreover, the first layer had 32 filters with an 8 × 8 kernel size.

There were two additional hidden Conv2D layers with 64 filters each, and a kernel size of 5 × 5 and 3 × 3, respectively. The final layer of the model was a dense layer consisting of 4 outputs, each output represented normal data or one of the 3 types of seizure. After optimization, the total number of epochs to train the network considering the size of EEG data was tuned to 20, and the learning rate was set at 0.001.

### 3.4. Extremely Random Tree Algorithm for Epileptic Seizure Detection

The extremely randomized trees approach, also known as extra-trees and was introduced in 2006, is an ensemble methodology that employs a large number of decision trees (DT) [44]. The ensemble approach is widely used in a variety of classification and regression tasks. The ensemble technique’s concept is to aggregate the decisions of several models and produce a decision based on that combination, which ultimately results in higher performance compared to that of a single decision or model. The DT-based ensemble technique can achieve better performance through randomization, provided the base learners are independent. Randomization improves the diversity of the trees and makes it easier to reduce correlation when developing trees. The ensemble learning approach works on the divide-and-conquer principle (or collective wisdom) to improve performance. In supervised machine learning tasks, an ensemble technique may be used to build a more stable and robust classifier (model) with precise predictions, minimizing factors such as noise, bias, and variance. However, the use of ensemble learning can be relatively expensive in terms of computational complexity due to the need to train a number of individual classifiers.

The ERT method consists of a number of DTs, each with a root node, child or split nodes, and leaf nodes. At the root node of a dataset, ERT chooses a split rule based on a random selection of characteristics and a somewhat predictable cut point. Each child node undergoes the same method until a leaf node is reached. The number of trees in the ensemble, the number of attributes/features to chosen randomly, and the minimum number of samples/instances necessary to divide a node are the three key parameters of ERT.

The ERT method is similar to the conventional random forest in that it is an ensemble of individual trees; however, there are two main distinctions. To begin, instead of training a bootstrap sample, each tree is trained using the whole learning sample. Secondly, the tree’s top-down splitting uses entirely random splits rather than the optimum splits. Instead of determining the locally optimal cut-point for each attribute being evaluated, based on Gini impurity or information gain, a random cut-point is employed. This point is chosen from the training set’s samples with a uniform distribution from the domain of the feature. Following that, the split with the highest score among all the randomly produced splits is chosen to split the node. The ERT method makes it significantly faster to train than a regular random forest approach as calculating the optimal split at each node for each feature takes a long time when constructing a DT. ERT also performs better compared to a random forest when there are instances with high noise in the data, which is common with sensors.

Moreover, when a test sample is passed through each DT and child node, the best splits are selected and the test sample is passed to the right/left child node until it reaches a leaf node. The leaf node in any DT determines the class for the test sample, and a majority of votes in the *k* decision trees decide the final prediction of the ERT algorithm.

The aggregate of unique errors, i.e., bias and variance, is the generalization error of the ML model. Underfitting, which may be computed as the capacity to correctly generalize unseen data, can be caused by a high bias. In other cases, a high variance can lead to overfitting, which is caused by the model’s high sensitivity to minor deviations in the training data. The ET algorithm reduces bias and variance error more effectively than any other randomization approach, such as a random forest. The cut-point selection and explicit randomization of the subset of characteristics reduce the variance, while the entire usage of the original training set to learn the individual DT reduces the bias.

Furthermore, one of the key benefits of ERT during implementation is that a great deal of focus is not necessary while selecting hyperparameter values. As the ERT model is so resistant to noise from a single DT, pruning is rarely necessary. In practise, the number of trees is regarded as the only parameter that must be addressed while building the ET model.

In this work, we implemented an ERT algorithm to detect seizure episodes using the EEG data as a benchmark for comparison with the performance of the RNN. Primarily, batch-mode operation was considered, which is based on a supervized algorithm that considers the machine learning problem presented by the total number of input values and one of its associated target values. The algorithm started with the addition of extra or additional trees using systematic empirical evaluation.

### 3.5. Enhanced ResNet Tuned for Epilepsy Detection

**Figure 3:**
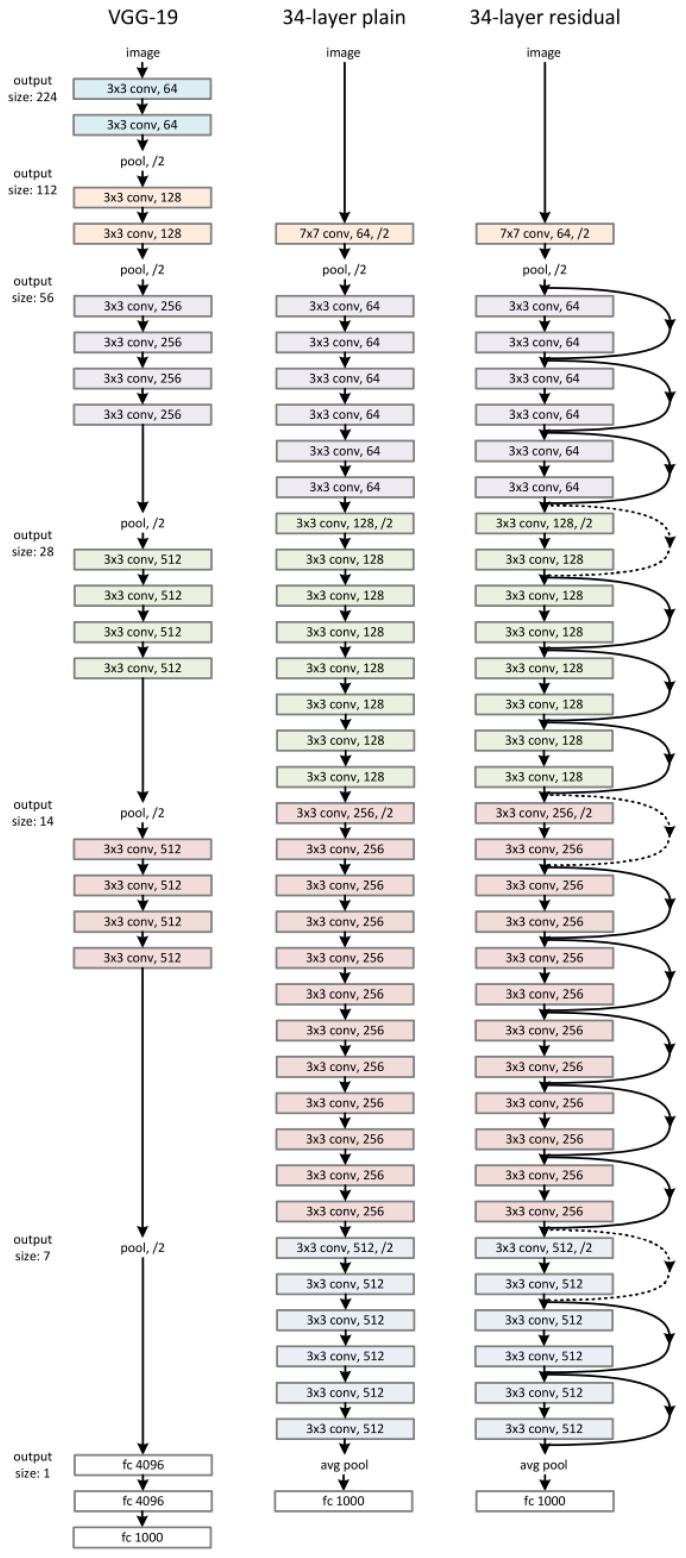
The architecture of the enhanced ResNet algorithm used in this work for epilepsy detection.

ResNet is a type of deep-learning algorithm that has been used in various applications, especially in the healthcare sector for monitoring patients through data acquisition and data analysis [45]. This work essentially utilized the enhanced residual convolutional neural network to identify epileptic seizure episodes exploiting EEG sensor data. A novel approach known as skin connection was used to train and validate the network model. The input layer that was used to give data, was utilized to feed the output higher layer to stack up the layers. The aim was to train the specific classification model to obtain the optimized target function values, such as f(x). In case the ResNet input and output were linked together, such that link connected was formed, the classification network model was generated to produce the model h(x)=f(x)−x instead of f(x). This state is known as residual learning [46]. The conventional deep neural network was initialized using zero weights, thus presenting zero values at the output side as well. On the contrary, the skip connection method introduced in ResNet presented similar values at the output side when fed at the input side, known as the objective function that accelerated the training and testing process. The skip connection method, in tandem with the objective function, efficiently traversed the EEG signals for the entire network. This work exploited the hyper-parameters to train the enhanced ResNet algorithm to detect epileptic seizure episodes as indicated in Table 2. The typical enhanced ResNet architecture used in this work is described in Figure 3.

As the deep neural network algorithms, such as the enhanced ResNet algorithm, improved their specific classification structure became deeper and deeper with the addition of extra layers; the deep network layers boosted the performance of complex feature extraction because of the “gradient explosion” that accommodated the limitations and time complexity of the conventional deep network algorithms, including taking a longer period of time to train the specific classification algorithm (time complexity), which caused the convergence to be more challenging or non-convergent. Additionally, the deep network algorithm’s performance accuracy, precision, F-measure and kappa values started saturating gradually due to degradation. To resolve both these issues that were encountered in the classification algorithm, we introduced very deep layers, i.e., 1000 layers, as indicated in Figure 3. This indicated that the identity mapping in the ResNet algorithm induced the changes in the output $F_{c}$ to a function value known as $F_{c}+x$. The training error of the enhanced ResNet algorithm, as derived, was relatively higher than that of the traditional convolutional networks. On the contrary, introducing various layers to the network mapping to the traditional convolutional network, such that the values of x=y, transformed the deep neural network algorithm’s layers and presented similar training error values. This mathematically indicated that the constant mapping introduced in the enhanced ResNet algorithm would perform better in terms of time complexity and hidden layers. When the enhanced ResNet algorithm’s values were 0, the stacking layer (1000) introduced would keep the network stable and constant when a large amount of EEG data were fed.

### 3.6. Feature Extraction for Enhanced ResNet Algorithm

In general, when extracting features from each channel from the ResNet algorithm, the time scalar values were required to estimate the weight values right before the scalar function values, known as global average pooling (GAP). The GAP method used in this work simplified the de-averaging of the discarded values obtained using the specific algorithm. To obtain the adequate amount of features from the diversity method of each ResNet layer/channel, the GAP was provided as the most feasible way to obtain the discrete function values and generalize the method across all used EEG data. Mathematically, it can be written as follows:

(10) att=sigmoid(fc(gap(X)))

Here the att values show the vector, the sigmoid values are the function for the sigmoid, the values contained in variable f and variable c are the mapping values of the enhanced ResNet algorithm, and gap indicates the GAP as discussed above. As soon as the vector values were received, each layer among the 1000 ResNet layers was scaled up by the associated elements using the channel retention method as follows:

(11) $$X_{:}^{*},i:,:={\mathrm{att}}_{i}X:,i,:,:,\;\;s.t.\;\;i\in 0,1,\ldots ,C-1$$

In Equation (11), $X^{*}$ shows the attendion method, att_*i*_ is the *i*th value of the vector in Equation (11), and X is the *i*th channel of the EEG input values. This is further elaborated in Equation (12):

(12) $$f_{k}=\sum_{i=0}^{L-1}x_{i}\cos\left(\pi k/L\left(i+1/2\right)\right),\;\;s.t.\;\;k\in 0,1,\ldots ,L-1$$

In Equation (12) the value of *f* indicates newly acquired values and *x* are the EEG data after pre-processing and can be written as follows:

(13) $$\begin{array}{c} x_{i,j}^{2d}=\sum_{h=0}^{H-1}\sum_{w=0}^{W-1}f_{h,w}^{2d}\cos\left(\pi h/H\left(i+1/2\right)\right)\cos\left(\pi w/W\left(j+1/2\right)\right)\\ \;s.t.\;\;i\in 0,1,\ldots ,H-1,j\in 0,1,\ldots ,W-1 \end{array}$$

As indicated in Equation (13), the mathematical modeling for the feature extraction and ResNet algorithm was dependent on the GAP function introduced earlier and also the sum of the weight of all 1000 layers used in the specific algorithm.

## 4. Results and Discussion

**Figure 4:**
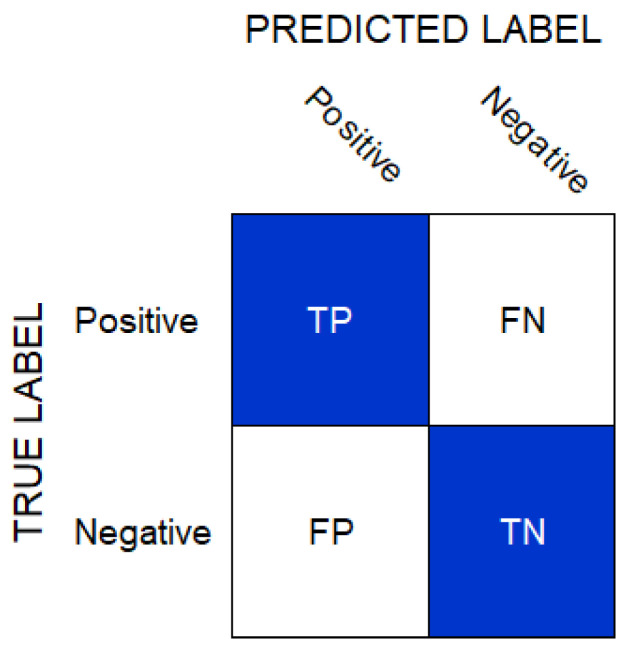
TP, TN, FP, and FN illustration using a confusion matrix.

This section presents the experimental results and discussion about the RNN-based, CNN-based, ERT-based, and ResNet-based models adapted for epilepsy seizure episode detection. The performance evaluation parameters included the *True Positives* (TP), *True Negatives* (TN), *False Positives* (FP), and *False Negatives* (FN). The TP represented the number of correctly identified positive instances whereas TN was the number of correctly identified negative observations. The FP was the number of positive predictions where the actual input was negative and, similarly, the FN showed the number of falsely negative predicted instances. These measures were presented in a confusion matrix, given in Figure 4.

The results were presented in commonly used performance metrics given by Equations (14)–(17).

(14) $$\;\mathrm{Accuracy}\;=\frac{\;\mathrm{TP}\;+\;\mathrm{TN}\;}{\;\mathrm{TP}\;+\;\mathrm{TN}\;+\;\mathrm{FP}\;+\;\mathrm{FN}}$$

(15) $$\;\mathrm{Precision}\;=\frac{\;\mathrm{TP}\;}{\;\mathrm{TP}\;+\;\mathrm{FP}\;}$$

(16) $$\;\mathrm{Recall}\;=\frac{\;\mathrm{TP}\;}{\;\mathrm{TP}\;+\;\mathrm{FN}\;}$$

(17) $$F1-\mathrm{score}=2\times \left(\frac{\;\mathrm{Recall}\;\times \;\mathrm{Precision}\;}{\;\mathrm{Recall}\;+\;\mathrm{Precision}\;}\right)$$

Before training any algorithm the data were shuffled to mix the observations from different classes. This technique usually reduces the model’s bias towards any class samples. After shuffling we first selected 90% of the data for training and the remaining 10% for testing. All the experiments were performed with the same training and testing data.

The ResNet algorithm was implemented using the Python tool in tandem with the NumPy libraries. The performance was given for identifying four classes of epileptic seizure using EEG signals. The grid search method was applied to obtain the optimal parameters of the algorithm during training where the total number of epochs was tuned to 20. The performance of the trained ResNet was evaluated in terms of accuracy, precision, and F-score values using test data. The model achieved a precision of 99.5%, F1-score of 96.3%, recall of 94.3%, and an accuracy of 96.1%.

**Figure 5:**
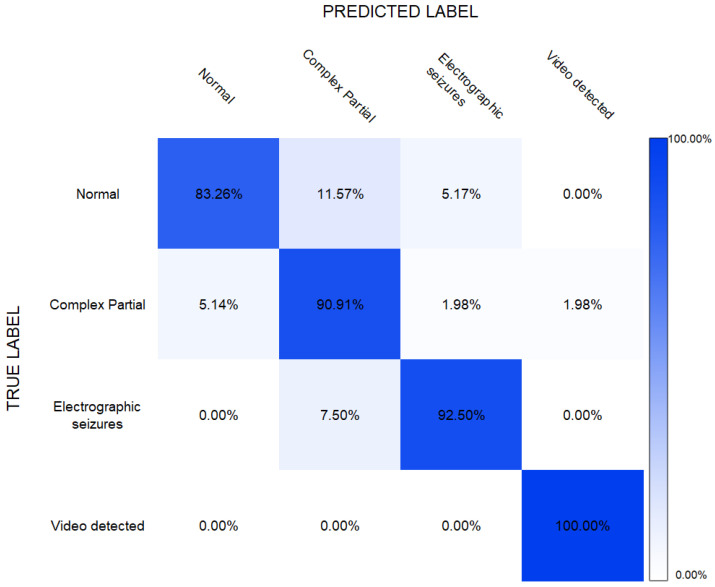
The ERT report in terms of a confusion matrix for four classes presenting epileptic seizures.

Figure 5 shows the confusion matrix of the results obtained from the ERT applied for the classification of four classes including “normal”, “complex partial seizures”, “electrographic seizures” and “video-detected seizures”. As shown, the normal, complex partial, and electrographic seizure classes were classified with respective accuracies of 83%, 90%, 92%, and the video-detected seizures with an accuracy of 100%. The highest number of mis-classifications in the complex partial class, of more than 11%, was noted when the normal class was given as input. On the other hand, more than 5% of actual complex partial classes were mis -classified as the normal class. These mis-classifications resulted because of high overlapping patterns among these classes. The least mis-classifications were seen between the normal and complex partial and electrographic seizure classes of about 1.98%. The point to be noted here is the classification in the case of the “video-detected” class, which surprisingly showed100% true classifications.

Furthermore, Table 3 depicts the ERT report based on 3 evaluation metrics: precision, recall, and F1-score. As can be seen, a score of more than 75% was secured by each metric for the normal and complex partial classes. Precision was highest reported with an almost 100% score for the electrographic seizures class. However, the score degraded slightly in terms of recall and F1-score for the complex partial and electrographic seizures classes. Nevertheless, the overall accuracy attained by ERT was 86%.

The results obtained by the CNN model are given in Table 4, showing the overall precision of 94.7% achieved by this model. The overall accuracy, recall, and F1-score were 94.0%, 94.1%, and 94.4%, respectively. Here, again, the precision, recall, and F1-score obtained resulted in 100% for the “video-detected” class. These results also showed that the classification of “complex partial” was comparatively challenging among all classes with a recall of 89.97% and an F1-score of 92.53%.

**Figure 6:**
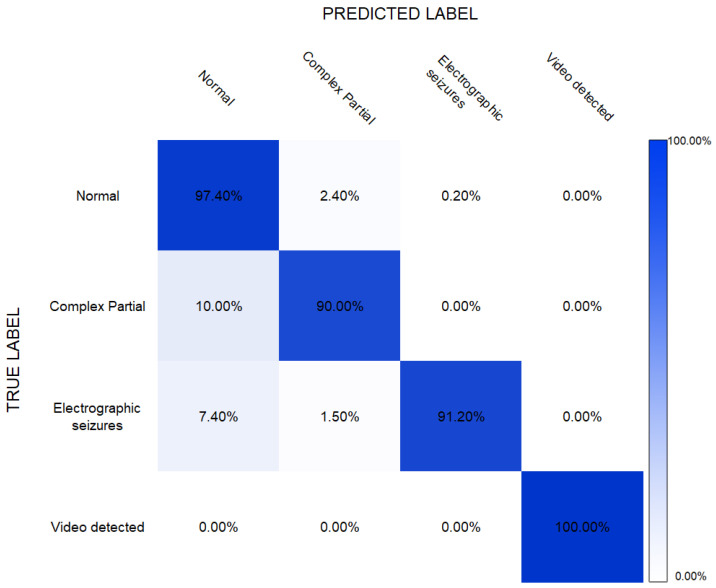
Confusion matrix for CNN-based epilepsy detection.

The confusion matrix shown in Figure 6 shows the accuracy of the CNN model achieved during the classification of the different classes, including normal and epilepsy seizures. It could be noted that the CNN model classified “complex partial seizures” with 90% accuracy, while “electrographic seizures” and “video-detected seizures” were classified with an accuracy of 91% and 100%, respectively. The normal signals were identified with 97.40% accuracy.

The RNN-based classification model was implemented using Matlab. The training of this algorithm took approximately 21 h with the system specifications of the AMD Ryzen 7 3700X processor and 16 GB RAM. After training, the model was used to classify unseen testing observations and the results are given in Table 5. These results showed that the RNN algorithm was able to achieve an accuracy of 97.6%.

The readings in Table 6 showed the results obtained after applying 10-fold cross-validation. It could be seen that the results were slightly improved; however, the difference was negligible with any variation in results being less than 0.5%.

**Figure 7:**
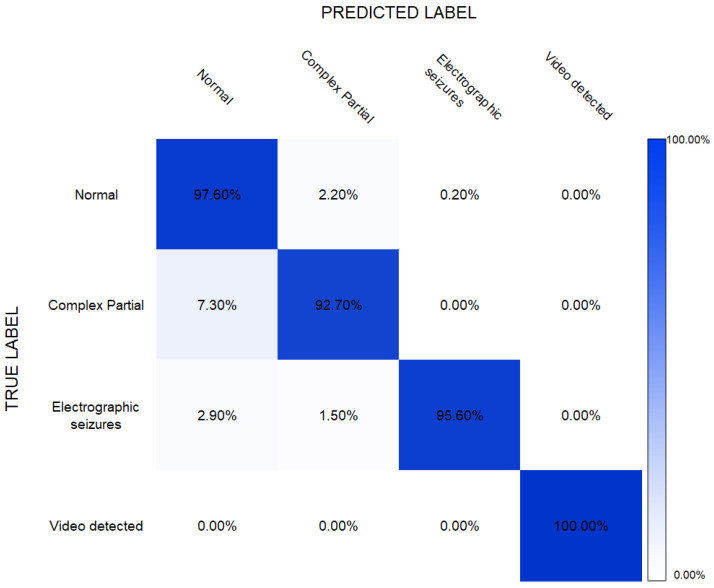
Confusion matrix for RNN-based epilepsy detection.

It can be seen that the RNN-based classification model could achieve a satisfactory performance of up to 91% and 96% accuracy when it came to the classification of “complex partial seizures” and “electrographic seizures”, respectively. The confusion matrix in Figure 7 shows the true positives, true negatives, false positives, and false negatives.

The advantage of RNN over other ML algorithms, including ResNet, ERT, and CNN, is that it requires less computational resources to predict the output, as is evident in our previous works [35,37]. It was estimated that the RNN required almost 50% of the computational resources than that required by a CNN with a similar number of layers while producing almost equal accuracy. Furthermore, the RNN had better generalization capabilities over other ML algorithms. The overall Accuracy, Precision, Recall and F1-Score is highlighted in Table 7.

## 5. Conclusions

In this work, a novel RNN-based epileptic diagnosis method was presented, and the application of various state-of-the-art machine learning algorithms to detect the different types of epileptic seizure episodes, which showed signs of confusion and loss of awareness using the electroencephalogram signals, was critically analysed. The machine learning algorithms used to detect the specific critical events included RNN, CNN, ERT, and ResNet, where four different classes of epileptic seizure were classified. Firstly, statistical features, such as standard deviation, kurtosis, skewness, and mean, were extracted from each EEG data channel containing 1 s of data. Secondly, the experiments were performed by using 90% of the data for training and the rest for testing purposes. Lastly, the ML models (RNN, CNN, ERT, and ResNet) were trained using features extracted from the aforementioned training data. The outcome concluded that the RNN algorithm provided the best classification accuracy of 97% without cross validation. However, after applying 10-fold cross validation, there was negligible improvement in the performance using RNN. Future work will evaluate the performance of these models under the unbalanced data problem between multiple classes.

## Figures and Tables

**Table 1:** Detail structure of the CNN architecture used in this research.

Type of Layer	Output Shape	Parameters
conv 2d (Conv2D)	(None, 12, 493, 32)	2080
dropout (Dropout)	(None, 12, 493, 32)	0
conv2d 1 (Conv2D)	(None, 8, 489, 64)	51,264
dropout 1 (Dropout)	(None, 8, 489, 64)	0
conv2d 2 (Conv2D)	(None, 6, 487, 64)	36,928
dropout 2 (Dropout)	(None, 6, 487, 64)	0
flatten (Flatten)	(None, 187,008)	0
dense (Dense)	(None, 32)	5,984,288
dropout 3 (Dropout)	(None, 32)	0
dense 1 (Dense)	(None, 4)	132

**Table 2:** Parameters used for training the deep ResNet algorithm.

Algorithm	Parameters
ResNet epochs	20
activation function	relu
optimizer	adam
loss	categorical-crossentropy

**Table 3:** Accuracy, precision, recall, and F1-Score obtained by the ERT model.

Type of Seizure	Accuracy	Precision	Recall	F1-Score
Normal (No seizure)	83.26%	81.26%	95.40%	89.52%
Complex Partial	90.91%	89.51%	79.24%	84.74%
Electrographic seizures	92.50%	92.84%	55.32%	67.31%
Video-detected with no visual change	100%	100.00%	13.23%	29.30%

**Table 4:** Accuracy, Precision, Recall, F1-Score and Accuracy obtained by CNN model.

Type of Seizure	Accuracy	Precision	Recall	F1-Score
Normal (No Seizure)	97.40%	93.75%	97.36%	95.52%
Complex Partial	90.00%	95.24%	89.97%	92.53%
Electrographic	91.20%	98.41%	91.18%	94.66%
Video-detected with no visual change	100.0%	100%	100%	100%

**Table 5:** Accuracy, precision, recall, and F1-Score obtained by the RNN model.

Type of Seizure	Accuracy	Precision	Recall	F1-Score
Normal (No seizure)	95.27%	93.76%	97.60%	95.64%
Complex Partial	95.65%	95.37%	92.73%	94.04%
Electrographic seizures	99.49%	98.48%	95.59%	97.01%
Video-detected with no visual change	100.0%	100.00%	100.00%	100.00%

**Table 6:** Accuracy, precision, recall, and F1-Score obtained by the RNN model after cross validation.

Type of Seizure	Accuracy	Precision	Recall	F1-Score
Normal (No seizure)	95.42%	93.78%	97.84%	95.64%
Complex Partial	95.91%	95.41%	93.43%	94.04%
Electrographic seizures	99.61%	98.51%	95.59%	97.01%
Video-detected with no visual change	100.0%	100.00%	100.00%	100.00%

**Table 7:** Overall Accuracy, Precision, Recall, and F1-Score.

Classification Method	Accuracy	Precision	Recall	F1-Score
ResNet	96.1%	99.5%	94.3%	96.3%
RNN	97.6%	96.9%	96.48%	96.7%
ERT	86%	96.4%	61.9%	70%
CNN	94%	96.9%	94.6%	95.7%

## Data Availability

The authors have used the publicly available Epileptic EEG Dataset. The dataset is available online https://data.mendeley.com/datasets/5pc2j46cbc/1 (accessed on 18 March 2022).
